# Are self-sacrificing employees liked by their supervisor?

**DOI:** 10.1007/s40821-023-00243-6

**Published:** 2023-04-26

**Authors:** Belén Bande, Takuma Kimura, Pilar Fernández-Ferrín, Sandra Castro-González, Abhishek Goel

**Affiliations:** 1 Faculty of Business Administration Studies, Universidade de Santiago de Compostela, Lugo, Spain; 2 Faculty of Lifelong Learning and Career Studies, Hosei University, Tokyo, Japan; 3 Faculty of Economics and Business Studies, University of the Basque Country UPV/EHU, Vitoria, Spain; 4 Indian Institute of Management Calcutta, Kolkata, India

**Keywords:** Impression management, Exemplification, Emotional intelligence, Liking, Performance, Socioemotional selectivity theory, M12, M31

## Abstract

Despite the growing prevalence of employee exemplification in the workplace, there is limited understanding of this assertive self-focused tactic. This study proposes to expand the exemplification research domain by exploring the emotional and behavioral conditions under which this impression management tactic is effective. Data analysis from 206 supervisor–employee dyads reveals that the indirect relationship between exemplification and individual performance through a supervisor’s liking is conditional on an employee’s emotional intelligence. Specifically, the exemplification effect on performance is sharply negative when a salesperson’s emotional intelligence is low, and it becomes insignificant when a salesperson is highly emotionally intelligent. This moderating effect is also strengthened by a supervisor’s age. Theoretical and practical implications are discussed.

## Introduction

“*I can do this job in fewer hours. But it will be seen as not giving the commitment. Being visible is a way of drawing attention to yourself. You are noticed more by being here at 10 at night than by consistently producing a good product*” (Rutherford, 2001, p. 273).

The above quote is from a senior executive who was describing employee exemplification, a self-focused impression management (IM) tactic that involves going above and beyond job requirements to gain recognition for being a dedicated employee (Jones & Pittman, 1982). The pressure to be an ideal worker is well established. The most desirable employee is the one who is always available for—and absolutely devoted to—their work (Acker, 1990). As Reid and Ramarajan (2016, p. 5) noted, “the expectation that people will be totally accessible and committed to work has never been stronger”. Flexible and remote work technologies and policies have also contributed to increasing the implicit expectation that employees will be available for work outside formal working hours (Arregui Pabollet et al., 2019). Given this context, presenting oneself as a role model is increasingly more frequent. For example, a study conducted by GfK Public Affairs & Corporate Communications and the US Travel Association in 2016 revealed that 22% of employees left some of their paid vacation time used to show complete dedication to their job.

Despite the growing prevalence of employee exemplification in the workplace and early acknowledgement that it is as likely to occur in an organizational setting as ingratiation (Feldman & Klich, 1991), researchers have paid little attention to exemplification (see Al-Shatti & Ohana, 2021; Bolino et al., 2008; Long, 2017 for reviews). Consequently, it has remained an understudied tactic (Bolino et al., 2008) despite the growing literature about IM in recent years (e.g., Boiral et al., 2020; Kibler et al., 2021; Peck & Levashina, 2017).

The current understanding of exemplification in an organizational setting is limited in at least two ways. First, studies that have examined the consequences of exemplification at work have arrived at inconsistent results regarding its impact on important work outcomes, revealing that strategic self-sacrificing behaviors can have both negative and positive consequences in terms of organizational outcomes (Bolino, 1999; Bolino et al., 2006, 2008; Harris et al., 2007; Liu et al., 2013; Wayne & Liden, 1995). Second, despite it being well known that social abilities such as self-monitoring or political skill determine IM configuration and effectiveness, there are still unanswered questions regarding why some people are better at managing impressions than others (Bolino et al., 2016; Maher et al., 2018). In particular, most social psychology and organizational behavior research has dealt exclusively with factors that operate at the actor level; even though it is understood that “when people manage their impressions in everyday life, they are usually engaged in an interdependent interaction in which actors and perceivers mutually influence one another” (Leary & Bolino, 2018, p. 259).

The present study’s purpose is to expand the exemplification research domain by exploring the role of exemplification on a salesperson’s individual performance ratings. Exemplification has been proposed as a kind of impression management tactic (Long, 2017). Employees often engage in such tactics to enhance their performance appraisals because the ratings employees receive play an important role in determining their worth to an organization (e.g., Bolino & Turnley, 2003; Bolino et al., 2006; Ferris et al., 1994). In line with previous research (e.g., Bolino et al., 2006; Wayne & Ferris, 1990; Wayne & Liden, 1995), it is postulated that impression management behaviors influence outcomes primarily through an affective response (Bande et al., 2017; Wayne & Liden, 1995). Liking is an important affective group cohesiveness component (Mullen & Copper, 1994), and it frequently influences important job outcomes (e.g., Allen & Rush, 1998; Wayne & Ferris, 1990). A recent meta-analysis suggested that “liking plays a role in facilitating relationship quality between supervisors and subordinates” (Dulebohn et al., 2017, p. 150).

**Fig. 1:**
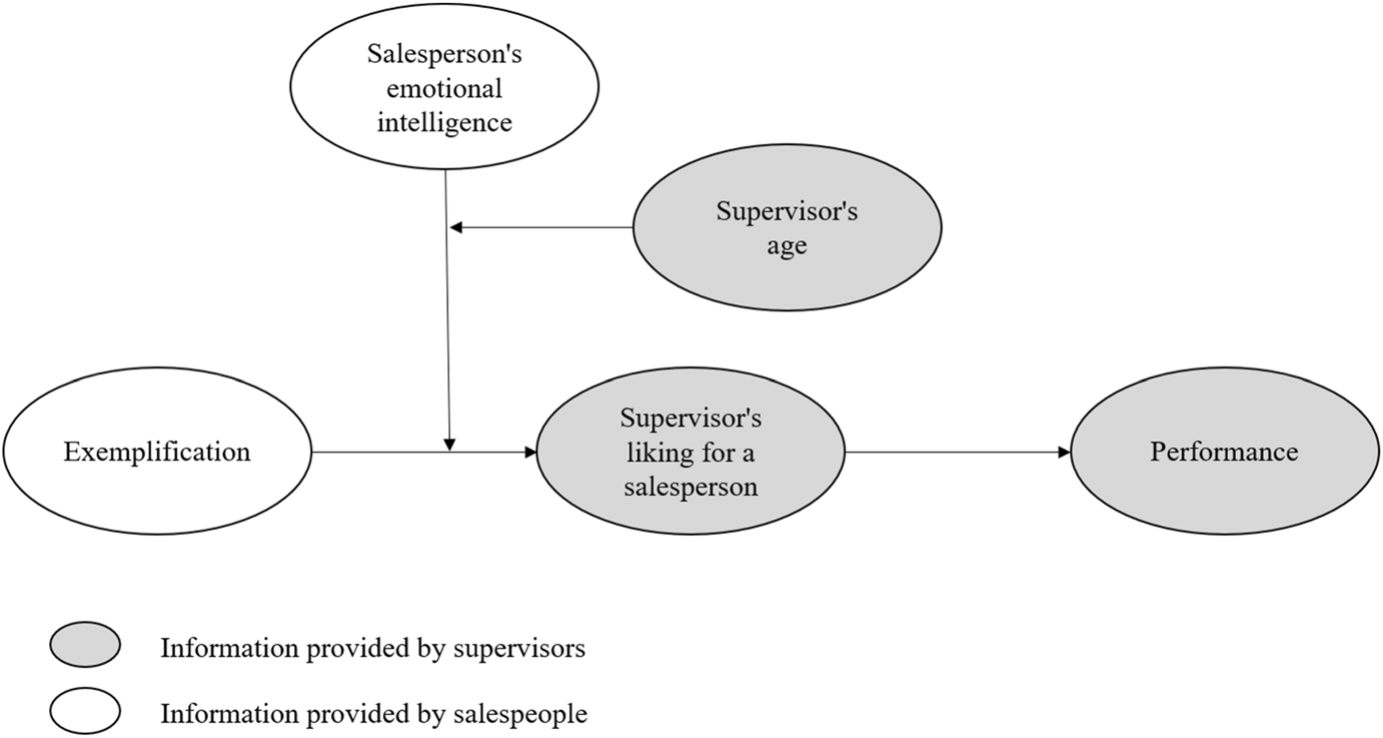
Proposed model

As shown in Fig. 1, this study examines the effectiveness of exemplification in terms of enhancing performance appraisals through supervisor’s liking, depending on the employee’s ability to manage their own emotions and to sense others’ emotions properly. In this respect, previous studies state that the relationship between exemplification and performance is inconsistent, suggesting the presence of mediating variables (Crawford et al., 2019). Similarly, Harris et al. (2007) found that exemplification was not a significant antecedent of supervisor appraisal of job performance, while Bande et al. (2017, p. 362) concluded that the effect of impression management behaviors on sales performance appraisal “is not a direct but an indirect one, through the impact of these tactics on the supervisors’ liking of the salesperson”. Building on this previous evidence, the direct impact of exemplification on individual performance was not included in the conceptual model.

Moreover, the influence of a target’s demographic characteristics (supervisor’s age) on exemplification effectiveness is also investigated. Individuals who have high emotional intelligence are aware of their own feelings and those of others. Such awareness enables them to regulate their and others’ emotions effectively (Wong & Law, 2002). Such sensitivity and regulation can be considered to enhance the effectiveness of impression management behavior, because the success of impression management depends on interpersonal interaction involving emotional arousal and expression.

Building on Long’s (2017, p. 49) suggestion that emotional intelligence (EI) is likely “another social skill that offers exemplifiers a greater chance at drawing desired impressions”, it is important to test whether emotionally intelligent individuals have a greater chance of success when using exemplification. Besides, it has been suggested that demographic characteristics are likely to influence the effectiveness of IM tactics (Bolino et al., 2016). Relying on socioemotional selectivity theory (Carstensen et al., 1999), this study surmises that an individual’s age affects how they perceive other people's behavior. As such, the moderating role of a supervisor’s age in the effectiveness of a salesperson’s use of exemplification tactics is examined.

In sum, the current study makes several important contributions to the literature. First, researchers have surprisingly paid very little attention to exemplification (Long, 2017). Moreover, results are very inconsistent regarding the positive, negative, or neutral effect of exemplification on work performance (e.g., Bande et al., 2017; Bolino et al., 2006; Higgins et al., 2003; Liu et al., 2013; Wayne & Liden, 1995). This work enhances understanding of the consequences of exemplification at work by determining what differentiates favorable target reactions to exemplification from unfavorable ones.

Second, this study recognizes that exemplification can be a risky strategy if it is not executed properly. This study proposes that the effectiveness of exemplification depends on an actor’s EI. Although EI has been regarded as the ability to be effective in social interaction (Long, 2017), its effect on impression management effectiveness has not been empirically tested. While previous studies have examined EI’s moderating effect as an antidote to personal threats (e.g., Ma & Liu, 2019; Szczygiel & Mikolajczak, 2018), they have paid scant attention to the proactive aspects of the EI role. Therefore, this study makes a novel contribution, shedding light on EI’s proactive aspects by revealing whether EI enhances the effectiveness of an actor’s proactive behavior, such as exemplification.

Third, most research on the consequences of IM tactics has dealt exclusively with factors at the actor level (Chawla et al., 2021; Crawford et al., 2019). However, focusing exclusively on an actor does not allow adequate assessment of the effectiveness of IM attempts (Kacmar & Carlson, 1999). This study examines a supervisor’s demographic characteristic, i.e., age, as a moderator that affects exemplification effectiveness. As such, this paper not only introduces the emotional aging perspective (i.e., Carstensen, 1992; Carstensen et al., 1999) in the study of IM in a work setting, but also responds to research calls for more attention to age as a moderating variable in organizational research (e.g., Ng & Feldman, 2010).

Finally, this study’s focus on salespeople can contribute both to sales management and to IM research in organizations in general. For many organizations, sales performance is considered critical for return on investment (Khusainova et al., 2018) because the sales function usually represents the largest proportion of a marketing budget (Cravens et al., 1993). Besides, previous studies have revealed that salespeople find themselves in situations at work where they can use influence tactics (Evans et al., 2012), and the spatial distance between salespeople and their supervisors allows greater opportunity for employing IM tactics such as exemplification (Ferris & Judge, 1991). Therefore, salespeople are relevant subjects for examining the effect and boundary condition of IM tactics.

This paper is structured as follows. First, we conduct a thorough review of the literature regarding the variables included in the study. Second, we formulate the hypotheses, and describe the data collection process and validation measures. Third, we present the results and discuss their theoretical and practical implications. Finally, we identify the main limitations of the study and outline future research opportunities.

## Literature review

### Exemplification, supervisor liking, and performance appraisal

Exemplification is a self-focused IM tactic that refers to behaviors individuals display to be seen as committed or hardworking (Bolino et al., 2008; Jones & Pittman, 1982). Identified as an assertive IM tactic employed to boost one’s image (Tedeschi & Melburg, 1984), exemplification is used by individuals to underscore their moral and social worthiness in order to garner the respect and admiration of the target of these actions (Cuddy et al., 2008; Jones & Pittman, 1982).

The literature has conceptualized an exemplifier as “the martyr who sacrifices for the cause” (Jones & Pittman, 1982, p. 244) in an endeavor to be recognized by others as dedicated, moral, and generous by demonstrating self-sacrificial behaviors that go above and beyond the call of duty (Long, 2017). In the employment context, these behaviors include working extra hours, taking shorter breaks, putting in extra effort, and displaying enthusiasm for their duties (Bolino & Turnley, 2003; Long, 2017). The ultimate reason for an individual’s use of exemplification tactics is to make a good impression and secure a positive outcome.

Previous studies have found that exemplification relates to performance appraisal, which determines one’s worth as an employee (Barrick et al., 2009; Bolino & Turnley, 2003; Bolino et al., 2006; Ferris et al., 1994; Wayne & Liden, 1995). Regarding the nature of this relationship, Crawford et al. (2019), building on self-verification theory, confirmed that exemplification rated by the subordinate was not significantly related to performance rated by the supervisor, suggesting the presence of intermediate variables in the relationship. Similarly, the affect-consistency bias theory, according to Guo et al. (2021), serves as a theoretical basis to suggest that supervisor’s affect towards the employee is key to the employee obtaining a positive performance appraisal by his supervisor when the employee performs exemplification behaviors such as working overtime.

Thus, consistent with previous research (e.g., Bande et al., 2017; Villanova & Bernardin, 1991; Wayne & Liden, 1995), this study expects exemplification to indirectly impact a supervisor’s rating of salesperson performance through the supervisor’s liking of the salesperson. A target’s emotion toward an agent has been the dependent variable in many studies on impression-management tactics (e.g., Bande et al., 2017; Bolino et al., 2006; Wayne & Liden, 1995).

Interpersonal liking is an emotional state created at the initial stage of an interpersonal relationship on the basis of personal preferences. However, liking constantly evolves and develops interactively (Zerubavel et al., 2018). Thus, a supervisor’s liking for their subordinates is susceptible to the subordinates’ IM. Although some literature conceptualizes exemplification as a self-focused, self-promotional IM tactic (Bolino et al., 2008), it can also be perceived as an attitude of a dedicated person. Accordingly, a subordinate’s exemplification can give a supervisor the impression that the subordinate values and respects their group, work role, and the supervisor’s instruction. Supervisors may tend to perceive that such subordinates are emotionally committed to their role and their supervisor. Besides, liking is an evolving process and is reciprocal—“individuals we like also like us, or vice versa” (Zerubavel et al., 2018, p. 4375). Therefore, a subordinate’s exemplification will enhance a supervisor’s liking for the subordinate.

Regarding the relationship between liking and performance, a supervisor’s affect toward a subordinate causes the supervisor to perceive and retain more positive performance-related behaviors, leading to a more favorable evaluation of a subordinate’s performance (Isen & Baron, 1991). Meta-analytic evidence confirms the long-standing belief that rater liking is positively related to performance ratings (Sutton et al., 2013). A more recent study also confirmed the positive effect of supervisor’s liking on performance appraisal (Bauch et al., 2021).

Thus, we hypothesize the following:

H1: Exemplification is indirectly related to individual performance through a supervisor’s liking for a subordinate.

### Moderating effect of emotional intelligence

Previous studies have also argued that self-focused IM tactics such as exemplification do not necessarily result in favorable outcomes for an agent, sometimes triggering negative target reactions (Powers & Zuroff, 1988; Wayne & Liden, 1995). These arguments suggest that exemplification is a complex tactic. In the use of exemplification, an actor runs the risk of failing to convey a positive image, appearing insincere, boastful, and too self-involved, thereby provoking a negative emotional response in an audience (Baron, 1986; Cialdini & De Nicholas, 1989). Wayne and Liden (1995) argued that self-focused IM tactics such as exemplification demand great skill. Their empirical analysis does not show a significant effect of subordinates’ self-focused IM on a supervisor’s liking.

An interpersonal influence model proposed by Levy et al. (1998) suggests that individuals who are skilled at engaging in IM behaviors are more likely to succeed using these tactics. Harris et al. (2007, p. 283) noted the importance of “measuring the skill of the influencer in determining if and why impression management behaviors lead to desired or undesired outcomes”. In this vein, self-monitoring and political skill are two social skills that decisively influence the effectiveness of impression-management tactics (Bolino et al., 2016; Turnley & Bolino, 2001).

In his theoretical review on exemplification in the workplace, Long (2017) asserted that the employee’s social skills play an important role in ensuring that these tactics lead to a positive result in terms of performance appraisal by the supervisor. Specifically, he draws attention to the need to explore the impact of the exemplifier’s emotional intelligence as an effective tool in interpersonal relationships. Similarly, taking a person-centered approach, Chawla et al. (2021) explored the consequences of combining positive and negative impression-management tactics. Their results confirm that it is necessary to nuance the consequences that these behaviors have on performance, pointing out the importance of the employee’s skills in obtaining a positive outcome.

Emotional intelligence has been suggested as another social skill that can help exemplifiers create the desired impressions in others (Long, 2017). Defined by Salovey and Mayer (1990, p. 189) as “the ability to monitor one’s own and others’ feelings and emotions, to discriminate among them and to use this information to guide one’s thinking and actions”, EI is a combination of cognition, emotion, and intelligence; it is considered as a critical determinant of workplace behavior (Winkel et al., 2011). In fact, there is a wide consensus on the role of EI as an antecedent of important work outcomes such as job performance, organizational citizenship behaviors, job attitudes such as job satisfaction, turnover intention, organizational commitment, leadership effectiveness, life satisfaction, stress, and work-family conflict (Bande et al., 2015; Miao et al., 2017; O’Boyle et al., 2011; Van Rooy & Viswesvaran, 2004; Walter et al., 2011).

An interesting issue related to current EI knowledge is the distinction between self-focused EI and other-focused EI (Pekaar et al., 2017; Salovey & Mayer, 1990). EI implies dealing with one’s own and others’ emotions, i.e., a dyadic exchange where the focus is on the self as well as the target’s emotional states. The influence of both dimensions can be found in distinct life domains (Pekaar et al., 2017). Being effective in dealing with others’ emotions was identified as an other-focused EI dimension by Salovey and Mayer’s (1990) model. It is called “identification of emotions” in their model and Riggio (1986) referred to it as emotional sensitivity. Emotional sensitivity is defined as the “process by which individuals receive and interpret the communicated messages of others” (Reichard & Riggio, 2008, p. 516). To achieve their attribution goals, exemplifiers engage in behaviors that they think will help their audience perceive them in a way that is consistent with their desired identity. To this end, exemplifiers must make predictions about how their target audiences will perceive them based on the acts they perform (Schneider, 1981). The process, therefore, involves interaction where an audience’s reaction to an actor serves as a cue for the actor to adjust their behavior as needed (Johnson et al., 2016).

An additional challenge during exemplification is to prevent an audience from believing that there is some hidden agenda (Ham & Vonk, 2011). If a target interprets a self-presentation attempt as insincere, it will backfire (Wortman & Linsenmeier, 1977). As Jones and Pittman (1982, p. 245) noted, “for appropriate social effect, the individual must exemplify morality and not merely claim it”. People with high emotional sensitivity decode others’ emotions quicker and anticipate others’ reactions to their behavior more accurately (Austin, 2004). The skillful appraisal of others’ emotions enables individuals to estimate others’ affective responses and to adapt their behaviors accordingly. Consequently, the behavior of those individuals can be perceived as authentic and friendly (Salovey & Mayer, 1990). In contrast, when an exemplifier fails to be perceived as authentic, audiences will hold negative impressions of the exemplifier because they perceive an ulterior motive behind the act (Fein et al., 1990).

Individuals with a high level of EI are also adept at emotional self-regulation (Salovey & Mayer, 1990). The effectiveness of exemplification may depend on an actor’s ability to manage emotions. Exemplifiers need to invest a lot of time and effort to go above and beyond their job requirements. To manage others’ impressions, exemplifiers need to pay attention to how their behavior will be perceived by their audiences. Moreover, they must also calibrate their behavior based on how they perceive their audiences’ reactions. Therefore, exemplification as an IM tactic is associated with an actor’s physical and cognitive burden.

Previous studies have revealed that experiencing work overload results in negative emotions and negative psychological states such as cynicism, emotional exhaustion, and anger (Greenglass et al., 2003; Grobelna, 2021). Negative affect can lead to self-focused attention, which reduces attentiveness to others. Moreover, through emotional contagion, actors’ negative emotion can lower the effectiveness of their social interaction. Thus, actors who are adept at managing their emotion may be better able to deal with the physical and cognitive burden associated with engaging in IM behavior. Their emotional management ability therefore contributes to maintaining or increasing the effectiveness of exemplification.

Based on these arguments, this study hypothesizes that exemplifiers’ emotional intelligence improves the influence of exemplifying behaviors on supervisor’s liking.

H2_:_ Salesperson emotional intelligence moderates the indirect effect of exemplification on individual performance through supervisor’s liking. The relationship will be stronger for salespeople with high EI and weaker for salespeople with low EI.

### Moderating effect of a supervisor’s age

This study includes supervisor’s age as another moderator of the main effect of exemplification on liking. Socioemotional selectivity theory argues that age is related to changes in motivation (Carstensen et al., 1999). The theory proposes that as individuals get older, they place greater emphasis on emotional goals rather than knowledge-related goals. Owing to this change in motivation, older people are likely to invest more cognitive resources to achieve emotional goals, which results in their biased attention to information that is relevant to their emotional goals. The theory also proposes that older people are likely to favor information that enhances their emotional satisfaction, exclusively because of their biased attention. This tendency is called “positivity effect” (Carstensen & Mikels, 2005).

SST suggests people’s worldviews change across their lifespan. Relying on the positivity effect, it has been suggested that as individuals get older, their well-being depends more on benevolence beliefs (Poulin & Silver, 2008). Thus, older people are likely to view the world as more benevolent. Empirical studies support this argument, showing that older adults perceived the world as more benevolent than younger adults (Calhoun et al., 1998; Poulin & Silver, 2008; Zacher & Froidevaux, 2021).

Relying on these arguments and empirical findings, this study proposes that the older the supervisor, the more likely it is that the subordinate’s exemplification enhances the supervisor’s liking for the subordinate. Exemplification is apparently a favorable behavior, but can be used as an IM tactic. Thus, some audiences can form a negative impression, suspecting an actor’s unfavorable ulterior motives. However, due to the positivity effect, older supervisors are more likely to regard subordinates' exemplification as a positive attribute.

Workplace communication is becoming more dependent on digital technologies such as emails, messaging tools, and video meetings. In line with this trend, IM is increasingly implemented via digitalized communication (Al-Shatti & Ohana, 2021). Recent studies show that SST also holds for digitalized communication, demonstrating that older people are likely to positively perceive their experience in digitalized interaction (Chan, 2018; Stevic et al., 2021). Correspondingly, empirical research on fake news sharing suggests that older people are more susceptible to manipulation by digitalized messages (Brashier & Schacter, 2020).

Empirical studies have also shown evidence of greater emotional expression in older people. For example, Malatesta-Magai et al. (1992) found that older individuals were more emotionally expressive than younger ones. Similarly, Dahling and Pérez (2010) confirmed that age was positively related to the expression of naturally felt emotions. It is therefore expected that exemplifiers with a high EI level will influence their supervisors’ emotions more effectively and quickly when a supervisor is older, leading to greater success of the exemplification attempt.

Consequently, this study proposes that the role of a salesperson’s emotional intelligence in obtaining a favorable performance appraisal using exemplification tactics will depend on a supervisor’s age. As a supervisor’s age increases, their emotions become more salient and easily observed by a salesperson. This study thus hypothesizes a three-way interaction between exemplification, actors’ emotional intelligence, and targets’ age.

H3: The moderating effect of emotional intelligence on the relationship between exemplification and sales performance appraisal will depend on a supervisor’s age. The effect will be stronger (more positive) as a supervisor’s age increases.

To summarize, relying on theories of self-verification and affect-consistency, previous findings suggest the existence of mediators in the relationship between exemplification and performance ratings (Crawford et al., 2019; Guo et al., 2021). However, prior studies have not identified the significant mediator, and, thus, the mechanism of the exemplification has not yet been sufficiently understood. Research on impression management has proposed and examined the moderating effect of the actor's skill and behavior on the effect of impression management based on the interpersonal influence model (Levy et al., 1998) and person-centered view (Chawla et al., 2021). However, there has been scarce attention to the audience side in this context.

Our model makes two major contributions. First, our model explores the mechanism of exemplification effect on performance ratings by suggesting the mediating effect of audience liking. The mediation is proposed by integrating the previous empirical findings on the effect of self-focused impression management and the affect-consistency bias theory. Second, relying on theories of interpersonal influence (Levy, 1998) and socioemotional selectivity (Carstensen et al., 1999), our model proposes a moderated moderation on exemplification effect. Our model emphasizes the actor-audience interaction in interpersonal influence by focusing on both actor’s and audience’s factors: subordinates’ emotional intelligence and supervisors’ age. This perspective can expand our understanding of interpersonal influence, such as impression management with exemplification.

## Methods

### Data collection

Data were collected by surveying salespeople and their immediate supervisors who worked in multiple firms located in the north-west region of Spain. Firms invited to participate belonged to distinct industries and operated in business-to-business settings. A total of 105 enterprises from industries including telecommunications, manufacturing, financial services, wholesale, and construction accepted an invitation to participate in this study. A contact person (sales director/human resources director) in each firm helped to randomly select a sales manager (105 supervisors) and up to three subordinates (210 salespeople). For most of the firms (82%), the ratio was one supervisor to two salespeople. Surveys were conducted using paper-and-pencil questionnaires and face-to-face interviews. To prevent social desirability bias, the questions related to the use of IM tactics were self-administered. One supervisor questionnaire and four salespeople questionnaires were discarded due to missing data.

Thus, the final sample comprised of 206 employee-supervisor dyads consisting of 104 supervisors and 206 salespeople. Most of the salespeople were men (73.8%) with a mean age of 39 years (SD = 8.3), and an average tenure within the organization of 7.8 years (SD = 7.9). Among the supervisors, 84.6% were male with a mean age of 44.6 years (SD = 9.2) and mean tenure of 14.5 years (SD = 10.2).

### Measures

All measures used in this study were developed from previously published research, and all scale items used seven-point Likert-type scales (1 = *strongly disagree*; 7 = *strongly agree*). Spanish versions of the measures were created by following normally used back-translation procedures (Brislin, 1970).

#### Exemplification

The use of exemplification tactics was measured by Bolino and Turnley’s (1999) four-item scale, which is based on Jones and Pittman’s (1982) taxonomy. The salespeople indicated their likelihood of engaging in the selected self-sacrificing behaviors. The Cronbach’s alpha value was 0.890 and the composite reliability was 0.857.

#### Emotional intelligence

Eight items from the Wong and Law Emotional Intelligence Scale (WLEIS) (Wong & Law, 2002) were used to capture the “others-emotion appraisal” and the “managing of emotions” dimensions of EI. The information was provided by the salespeople. The Cronbach’s alpha value for the eight-item scale was 0.837 and the composite reliability was 0.893.

#### Affect

Supervisors’ affect toward the salespeople was reported using the three-item scale measure developed by Wayne and Liden (1995). The Cronbach’s alpha value was 0.892 and the composite reliability was 0.893.

#### Performance

Salesperson performance at an individual level was assessed using a nine-item scale developed by Griffin et al. (2007). The supervisors provided information about their subordinates’ individual task proficiency, adaptivity, and proactivity. The Cronbach’s alpha value was 0.908 and the composite reliability was 0.921. The supervisors’ age was measured in years.

The salesperson sales experience was controlled for by taking into consideration prior meta-analyses (i.e., Quiñones et al., 1995); the positive relationship between job experience and performance was confirmed. Salesperson gender was also controlled for because previous meta-analytic research on job performance ratings reports slightly higher scores for females than males (Roth et al., 2012).

Descriptive statistics, Cronbach’s alpha, and correlations are provided in Table 1.

**Table 1:** Means, standard deviations and correlations among variables

Variable	Mean	S.D	1	2	3	4	5	6	7
1. Exemplification	2.481	1.291	(0.601)						
2. EI	5.760	0.716	0.050	(0.512)					
3. Liking	5.496	1.158	− 0.148*	0.146*	(0.738)				
4. Performance	5.591	0.942	− 0.071	0.270**	0.649**	(0.57)			
5. Supervisor’s age	44.680	9.197	0.133	0.207*	− 0.042	− 0.031			
6. Salesperson experience	15.160	18.007	0.091	0.131	0.084	0.162*	0.204**		
7. Salesperson gender	1.260	0.441	− 0.118	− 0.005	0.145*	0.166*	0.018	− 0.001	–

**p < 0.01, *p < 0.05/Gender coded: 1 = male; 2 = female

AVEs appear diagonally

#### Measurement validation

The measurement properties of the multi-item scales used in this study were evaluated by maximum likelihood confirmatory factor analysis (CFA) using AMOS 24 (Gerbing & Anderson, 1988). The results suggested an acceptable fit: χ^2^ = 321.356; df = 235; p < 0.01; χ^2^/df = 1.367; RMSEA = 0.040; TLI = 0.961; IFI = 0.970; CFI = 0.968. All indicator loadings were significant (p < 0.01) and greater than the 0.50 value, thus supporting the validity of the items used in the study (see Appendix (Table 6)). In addition, the average variance extracted (AVE) and the composite reliabilities all exceed the commonly recommended thresholds (Bagozzi & Yi, 1988).

Fornell and Larcker’s (1981) approach was used to assess discriminant validity. The AVE for each construct exceeds the squared correlations between all pairs of constructs. Thus, discriminant validity between constructs was assumed.

## Results

### Moderated mediation effects

**Fig. 2:**
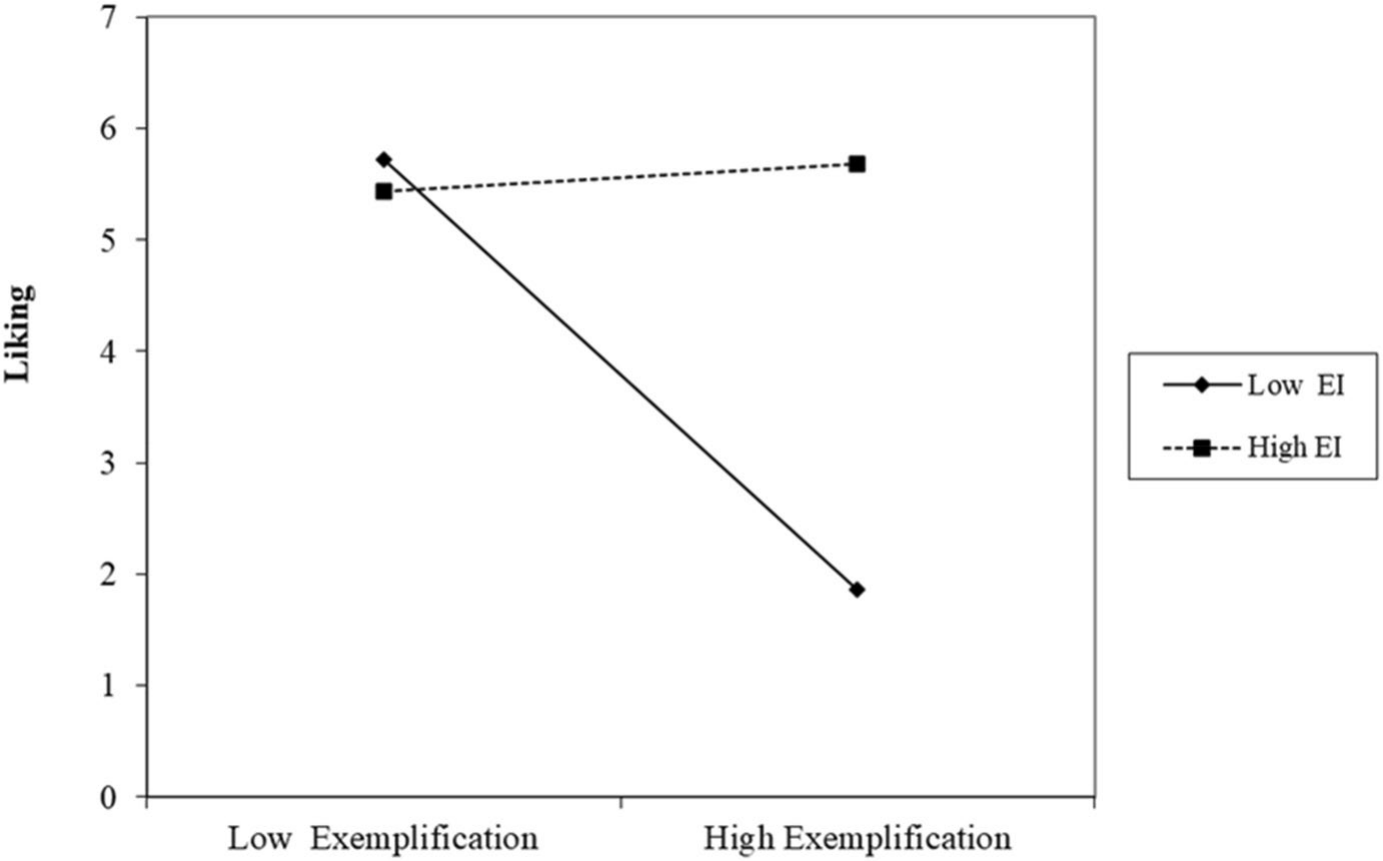
Moderating effect of EI

The moderating effect of EI on the indirect influence of exemplification on salesperson performance was examined using the bootstrap procedure prescribed by Hayes (2013) and Zhao et al. (2010). Salesperson sales experience and salesperson gender were included as control variables in the analysis. The moderated mediation analysis results (PROCESS Model 7; Hayes, 2013), using ordinary least squares (OLS) and mean-centered variables, are shown in Tables 2 and 3. The results show that exemplification is negatively related to supervisor’s liking (β =  − 0.170; p < 0.05; SE = 0.069), which in turn is positively related to performance appraisal (β = 0.753; p < 0.01; SE = 0.065). Moreover, EI moderates the association between exemplification and supervisor’s liking (interaction term: β = 0.216; p < 0.05; SE = 0.102). A simple slope diagram to visualize the patterns of this interaction (see Fig. 2) shows that the negative relationship between exemplification and supervisor’s liking is stronger (more negative) for employees with low EI (β =  − 0.672; p < 0.01) and weaker for salespeople with high EI (β = 0.043; n.s).

**Table 2:** Model coefficients for the conditional process analysis (moderated mediation)

Antecedents	Consequences					
	M(Liking)			Y(Performance)		
	Coeff.	SE	p	Coeff	SE	p
Constant	4.983***	0.246	< 0.001	3.646***	0.399	< 0.001
Exemplification	− 0.170*	0.069	< 0.05	0.024^ns^	0.057	0.66
EI	0.278*	0.110	< 0.05	–	–	–
Liking	–	–	–	0.753***	0.065	< 0.001
Exemplification x EI	0.216*	0.102	< 0.05	–	–	–
Salesperson experience	0.006^ns^	0.004	0.127	0.007^ns^	0.004	0.077
Salesperson gender	0.334^ns^	0.177	0.061	0.233^ns^	0.168	0.167
	R^2^ = 0,09, F(5,195) = 3.713, p = 0 < 0.01			R^2^ = 0,435, F(4,196) = 37.853, p = 0 < 0.001		

*p < 0.05; ** p < 0.01; *** p < 0.001; ns not significant

**Table 3:** Direct and indirect effects of exemplification on salesperson performance at values of EI (moderated mediation)

Conditional indirect effects of exemplification on individual performance through supervisor’s liking at values of emotional intelligence				
E. intelligence*	Indirect effect	BootSE	BootLLCI	BootULCI
4.750	− 0.293	0.122	− 0.553	− 0.068
5.250	− 0.211	0.087	− 0.397	− 0.050
5.750	− 0.130	0.059	− 0.257	− 0.019
6.250	− 0.048	0.052	− 0.156	0.052
6.750	0.033	0.072	− 0.119	0.168

Unconditional direct and indirect effects of exemplification on individual performance				
Direct effect	SE	P	LLCI	ULCI
0.024	0.057	0.664	− 0.088	0.138

*Values are for the 10th, 25th, 50th, 75th, and 90th percentiles

Thus, the indirect relationship between exemplification and performance appraisal is significant, although negative, and conditional upon a salesperson’s EI level. Specifically, in support of hypothesis 2, the negative link between exemplification and performance becomes weaker (less negative) as EI increases. The significance of the conditional indirect effect is also confirmed by the overall index of moderated mediation (Index = 0.163; SE (Boot) = 0.081; 95% Boot CI = 0.005; 0.326).

The significant moderated mediation model was further tested by examining the indirect effect of exemplification on performance appraisal at different EI levels (see Table 3). The pick-a-point approach results show the conditional effects of exemplification on performance appraisal through liking for the 10th, 25th, 50th, 75th, and 90th percentiles in the sample distribution of EI. When EI is very low (10th percentile), the indirect effect of exemplification on performance is − 0.293. This effect diminishes as EI increases, becoming equal to − 0.130 for values of EI on the 50th percentile. At high values of EI (above 5.75—75th and 90th percentiles) the indirect effect of exemplification on performance through liking becomes insignificant. Moreover, this effect is an indirect-only mediation because exemplification has no direct effect on salesperson performance appraisal (β = 0.024; SE = 0.057; p = 0.664; [− 0.088; 0.138]). Thus, hypothesis 1 is partially supported.

### Moderated moderated mediation effects

To test the moderating influence of a supervisor’s age, a three-way interaction model (Model 11; Hayes, 2013) was used, in which supervisor’s age operated as a secondary moderator of the indirect relationship between exemplification and performance appraisal (see Table 4). Salesperson experience and salesperson gender were included as control variables in the analysis. The moderated moderated mediation model showed that a three-way interaction between exemplification, EI, and supervisor’s age significantly affected supervisor’s liking (β = 0.026, SE = 0.012; p < 0.05). Moreover, the overall index of the moderated moderated mediation confidence interval did not straddle zero within its lower and upper limits (Index = 0.019; SE (Boot) = 0.010; 95% Boot CI = 0.003; 0.040), confirming the significance of the conditional indirect effect. Thus, hypothesis 3 was supported.

**Table 4:** Model coefficients for the conditional process analysis (moderated moderated mediation)

Antecedents	Consequences					
	M (Liking)			Y (Performance)		
	Coeff.	SE	*p*	Coeff	SE	*p*
Constant	5.035***	0.245	< 0.001	3.646***	0.399	< 0.001
Exemplification	− 0.169*	0.070	< 0.05	0.025^ns^	0.057	0.665
EI	0.278*	0.112	< 0.05	–	–	–
Liking	–	–	–	0.754	0.066***	< 0.001
Supervisor’s age	− 0.009^ns^	0.009	0.300	–	–	–
Exemplification × EI	0.233*	0.102	< 0.05	–	–	–
Exemplification × Supervisor’s age	− 0.005^ns^	0.008	0.559	–	–	–
EI × Supervisor’s age	− 0.018^ns^	0.012	0.136	–	–	–
Exemplification × EI × Supervisor’s age	0.026*	0.012	< 0.05	–	–	–
Salesperson experience	0.008^ns^	0.004	0.069	0.007	0.004^ns^	0.077
Salesperson gender	0.310^ns^	0.176	0.081	0.233	0.168^ns^	0.167
	R^2^ = 0.125, F(9,191) = 3.023, *p* < 0.01			R^2^ = 0.43, F(4,196) = 37.85, p < 0.001		

*p < 0.05; ** p < 0.01; *** p < 0.001; ns not significant

The pick-a-point approach results also show the conditional effects of exemplification on performance appraisal through liking for the 10th, 25th, 50th, 75th, and 90th percentiles in the sample distribution of EI and supervisor’s age (Table 5).

**Table 5:** Conditional indirect effects of exemplification on performance through liking at values of EI and supervisor’s age (moderated moderated mediation)

E. intelligence*	Supervisor age*	Indirect effect	BootSE	BootLLCI	BootULCI
4.750	33	− 0.034	0.156	− 0.371	0.254
4.750	37	− 0.126	0.129	− 0.411	0.105
**4.750**	**45**	− **0.312**	**0.124**	− **0.579**	− **0.090**
**4.750**	**50**	− **0.428**	**0.156**	− **0.768**	− **0.141**
**4.750**	**55**	− **0.544**	**0.201**	− **0.969**	− **0.168**
5.250	33	− 0.059	0.108	− 0.319	0.122
5.250	37	− 0.113	0.091	− 0.320	0.041
**5.250**	**45**	− **0.221**	**0.088**	− **0.414**	− **0.067**
**5.250**	**50**	− **0.289**	**0.108**	− **0.529**	− **0.097**
**5.250**	**55**	− **0.356**	**0.137**	− **0.659**	− **0.111**
5.750	33	− 0.084	0.081	− 0.263	0.059
5.750	37	− 0.100	0.067	− 0.243	0.020
**5.750**	**45**	− **0.130**	**0.060**	− **0.258**	− **0.025**
**5.750**	**50**	− **0.150**	**0.072**	− **0.316**	− **0.027**
**5.750**	**55**	− **0.169**	**0.091**	− **0.378**	− **0.011**
6.250	33	− 0.109	0.094	− 0.327	0.049
6.250	37	− 0.086	0.073	− 0.254	0.035
6.250	45	− 0.040	0.055	− 0.153	0.067
6.250	50	− 0.011	0.069	− 0.156	0.114
6.250	55	0.018	0.094	− 0.183	0.189
6.750	33	− 0.135	0.136	− 0.477	0.070
6.750	37	− 0.073	0.104	− 0.344	0.079
6.750	45	0.051	0.079	− 0.104	0.208
6.750	50	0.128	0.102	− 0.083	0.322
6.750	55	0.205	0.142	− 0.104	0.467

Bold values indicate significant indirect effects

*Values are for the 10th, 25th, 50th, 75th, and 90th percentiles

**Fig. 3:**
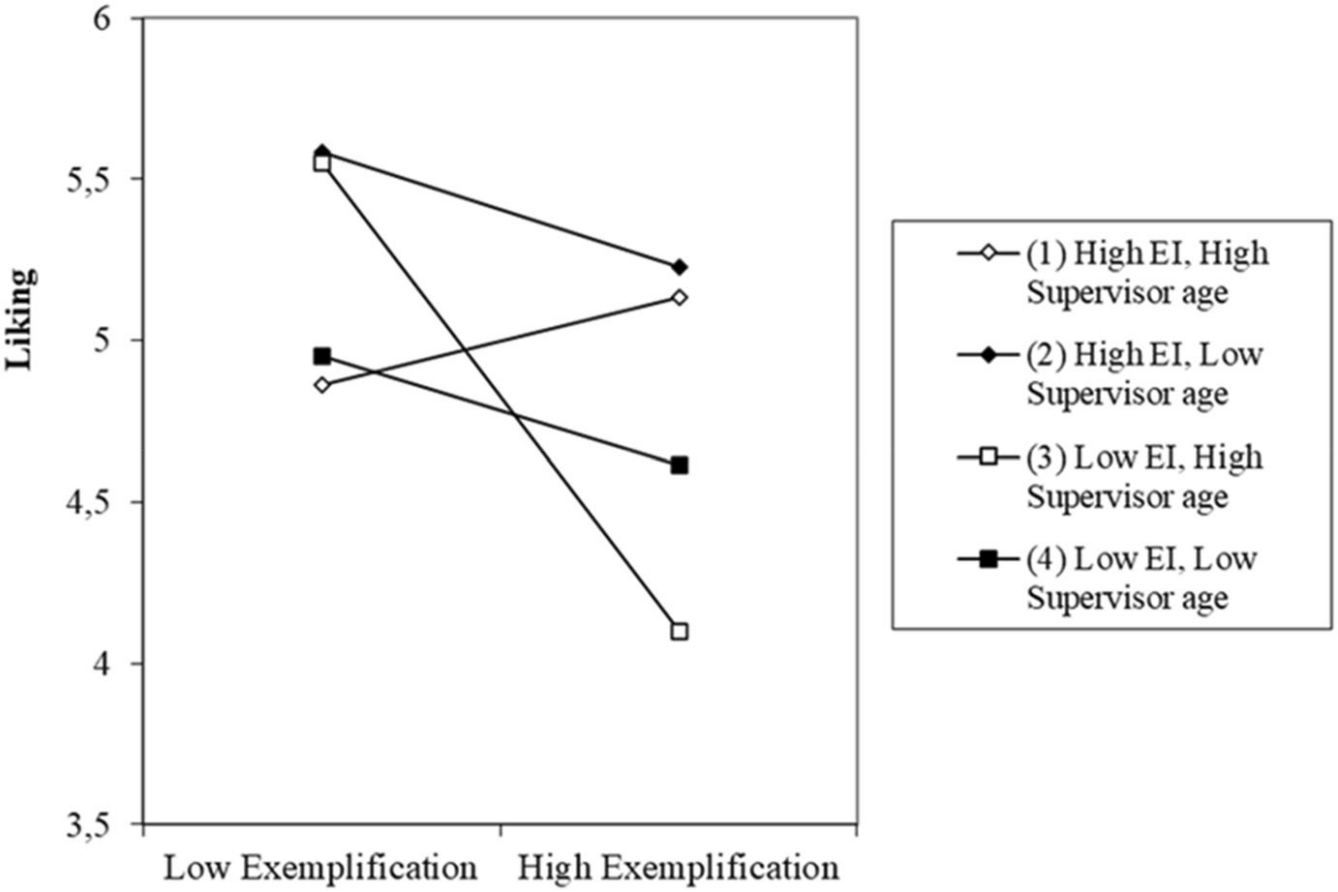
Three-way interaction (EI, supervisor age, exemplification)

Figure 3 displays the plots for the three-way interaction model. The effect of exemplification on supervisor’s liking is sharply negative for salespeople with low EI and those whose supervisor is older (slope 3). In contrast, the positive effect of exemplification on supervisor’s affect toward a salesperson occurs only when both EI and supervisor’s age have high values (slope 1). As predicted, the effect of exemplification on performance appraisal through supervisor’s liking depends on both the salesperson’s EI and on the supervisor’s age. Thus, as the age of a supervisor increases, the buffering effect of EI on the relationship between exemplification and performance appraisal becomes stronger. Moreover, as a supervisor ages, the exemplification effect combined with a salesperson’s ability to handle emotions changes from negative to positive.

## Discussion

### Theoretical implications

IM theory and research, which are not limited to exemplification, have paid little attention to emotion. This is surprising given “expressive behaviors have been considered a part of IM for decades” (Johnson et al., 2016, p. 118). Early IM studies recognized the importance of emotion regulation in impression management (e.g., Schlenker & Weigold, 1992; Weinberger et al., 1994). However, the effectiveness of sensing and regulating emotion in IM has been under-researched in empirical settings.

The purpose of this research was to expand the exemplification and IM tactics literature by delving into when and how self-sacrifice at work is effective. It was found that the effect of exemplification tactics varies across actors and targets. These findings join some recent studies (e.g., Brouer et al., 2015; Plouffe et al., 2014) that confirm, contrary to the traditional literature approach, that IM tactics do not follow a one-size-fits-all model but that their effectiveness depends on who uses them, how they are used, and toward whom they are directed.

Many employees assume that their supervisors value people who work long hours. Dedication and hard work are often praised and characterized as desirable moral values in others (Karlberg, 2002). Consequently, employees devote time and energy toward appearing dedicated, generous, and hardworking individuals in the workplace (Jones & Pittman, 1982; Wayne & Liden, 1995). For years, emotion researchers have drawn attention to the importance of strategic emotion displays when influencing others (e.g., Kopelman et al., 2006). However, the influence of emotional intelligence on the success of exemplification as an IM attempt is still understudied.

The findings of this study suggest that an employee’s successful presentation of themselves as an ideal worker depends on their EI. These results specifically show that actors’ adequate perception and interpretation of a target’s emotions are necessary to prevent an IM attempt from backfiring. At low levels of EI, the influence of exemplification on performance appraisal is negative. As emotional intelligence increases, the negative effect on performance appraisal becomes insignificant. However, inconsistent with this study’s expectations, even when EI is high, the relationship between exemplification and performance appraisal is still insignificant, not significantly positive.

One possible explanation is that exemplification is not very effective at influencing interpersonal liking. Presenting oneself as an exemplary and respectable person does not induce interpersonal liking. Rather than such self-focused IM tactics, target-focused tactics will be more effective. As suggested by previous studies, showing similarity with or liking for a target will more strongly [induce/influence] the target’s liking for an actor (Byrne et al., 1966; Condon & Crano, 1988; for review, Montoya et al., 2008). Another possible explanation is that job requirements for salespersons have become increasingly demanding. A certain level of exemplification is thus perceived as nothing more than an employee’s job duties. Accordingly, properly engaging in exemplification can only avoid creating an undesirable impression that results in a negative appraisal from supervisors.

These findings partially offer support for the notion that, in an interpersonal setting, “emotions have informational value for others” (Fischer & Manstead, 2008, p. 459). The ability of individuals to effectively perceive the emotions of those around them can ensure interpersonal cooperation and successful communication with others (Dubé et al., 1996). As Lopes et al. (2006) noted, emotional abilities may help people to communicate effectively and influence others to get what they want. The findings only partially align with the arguments of past researchers who have proposed EI as a key variable when adapting more effectively to the environment, taking into consideration that the ability to manage emotions may influence the use of effective interaction strategies (e.g., Furr & Funder, 1998; Langston & Cantor, 1989). As Pekaar et al. (2017) stated, when the objective is to achieve a social goal, focusing on others’ emotions can be particularly effective because it can help to influence their behavior and mood.

In sum, and consistent with past research, this study’s results indicate that efforts to be seen as likeable may backfire when people lack social skill (Harris et al., 2007; Turnley & Bolino, 2001). Harris et al. (2007) similarly confirmed that exemplification is positively related to performance when an actor’s political skill is high, but negatively related to it when their political skill is low. This study’s results confirm that EI is an additional social skill on which the success of IM tactics relies. The study’s findings endorse Long’s (2017) proposition that EI has an effect on exemplification, which has not been examined in empirical studies. Although recent studies have suggested the relevance of this variable in the context of exemplification tactics (i.e., Long, 2017), it has not been analyzed to date.

However, displaying EI may not attenuate the negative consequences related to individuals’ use of exemplification tactics. This study’s findings suggest that the success of an exemplification attempt not only depends on an employee’s emotional intelligence, but also on a supervisor’s age. As predicted, when a supervisor’s age increases, the effect of EI on the relationship between exemplification and sales performance appraisal is stronger (more positive). In fact, exemplification succeeds if both a salesperson’s emotional sensitivity and a supervisor’s age have high values. When a supervisor’s age is low, the exemplification effect on a supervisor’s liking is not conditioned by an employee’s emotional skills. As individuals age, they display higher levels of sympathy and emotion sharing (Richter & Kunzmann, 2011). Thus, employees can more easily and accurately perceive their supervisor’s emotions as their age increases, enhancing the positive effect of an employee’s emotional sensitivity on the exemplification-sales performance relationship. This study’s findings align with the notion that age is an important demographic characteristic that affects human resources decisions and actions (Ferris et al., 1991) and that the effective use of IM tactics is conditional upon the target audience (Liu et al., 2013). Consequently, this study also responds to recent calls for research on the effects of age across various aspects of work performance (e.g., Ng & Feldman, 2013), which is particularly relevant given the current workforce characteristics. The number of older workers in the labor force is currently rising in most industrial countries. In the EU, the number of people aged 55 years and over is predicted to increase by more than 15% between 2010 and 2030 (Schalk et al., 2010).

It should be noted that in the three-way interaction analysis, the most negative effect between exemplification and performance occurs for low values of EI and high supervisor age. Besides, as a supervisor’s age decreases, the positive EI effect becomes smaller. The fact that the largest negative effect was seen when low EI and high age setting were applied suggests that age does not simply hinder supervisors’ detection of IM. A possible explanation is that for subordinates with high EI, socioemotional selectivity theory applies; however, for those with low EI, SST does not apply and exemplification backfires. The results indicate that although older supervisors can detect poor exemplification attempts, they can be manipulated by tactful ones.

Contrary to the SST tenet, older supervisors might be adept at detecting IM owing to their long work experience. This study’s results suggest that SST does not always work in the proposed way. For SST to work, the stimulus to an older person should have a certain level of ambiguity. Specifically, the stimulus should be difficult to detect and interpret; otherwise, an older person’s experience overcomes the SST effect. Although these findings do not provide sufficient evidence to endorse these arguments, it paves the way for expanding future SST research.

This study’s findings bolster the arguments of past researchers who have suggested that employees must be willing to assume risk when using self-focused tactics given that an attempt at influence can backfire when a target interprets the behavior as deceitful (Liden & Mitchell, 1988; Wayne & Liden, 1995). There is also support for Villanova and Bernardin’s (1989) statement regarding the lack of guarantee that the use of IM behaviors will translate into increased liking.

The effect of exemplification on performance ratings is very inconsistent (e.g., Bolino, 1999; Bolino et al., 2006; Wayne & Liden, 1995). Moreover, studies have obtained inconsistent results regarding the relationship between self-focused IM tactics and a supervisor’s affect toward an employee (Bande et al., 2017; Bolino et al., 2006; Wayne & Liden, 1995). This study finds support for the mediating effect of a supervisor’s affect toward a subordinate in the relationship between exemplification and performance appraisal, which contributes to explaining the lack of support for a direct relationship found in some previous research (e.g., Brouer et al., 2015). It is also important to note that liking between a leader and a follower, unlike similar constructs such as leader-member exchange, does not need to be mutual (Dulebohn et al., 2017).

### Practical implications

Aiming at strengthening the connection between research and practice (Ratchford, 2020), this study provides important managerial implications. Sales managers are encouraged to be objective in their performance appraisals (Gentry et al., 1991). It is important that organizations have a real and accurate picture of their employees. Thus, excluding bias in the rating process is important because the appraisal should reflect an employee’s true performance (Yun et al., 2005). However, this study’s findings align with the idea that performance ratings may be distorted by IM tactics and individual characteristics (Brouer et al., 2015; Harris et al., 2007). Most employees use exemplification to some extent, and everyday practice makes the use of exemplification habitual (De Cuyper et al., 2014). Decision makers should be concerned about performance appraisal precision because it can be affected, either in a positive or negative way, by an individual’s effective use of IM tactics. This idea aligns with the notion that the motivation for using IM tactics is greatest when an evaluation event is near (McFarland et al., 2022). In this regard, some training techniques such as video modeling have been found to be effective for training supervisors to implement certain skills related to accuracy in performance procedures (Shuler & Carroll, 2019).

From an employee perspective, subordinates should be concerned about the potential negative consequences of engaging in self-sacrificial behaviors at work. Improving their emotional skills may be an effective way to prevent such behaviors backfiring on them. In this sense, there is evidence that EI can be developed through training (Luthans, 2012).

### Limitations and future research directions

Despite its significant contributions, this study is not without limitations. Following Bolino and Turnley’s (1999) recommendation, this study relies on salespeople’s self-reported IM behavior, as does much of IM tactics research. However, it would also be useful to obtain data from other sources, such as supervisors, coworkers, or external observers. This may permit researchers to identify those who can more accurately evaluate IM behaviors (Bolino et al., 2016). Moreover, the research design was cross-sectional—a longitudinal design is recommended to test the causal relationships.

This study’s findings suggest several interesting research directions. In addition to overcoming the above limitations, it would be useful for researchers to consider other understudied IM tactics, such as intimidation or supplication. It has also been suggested that trying to impress others by appearing to be a more dedicated employee may result in other employees looking less dedicated in contrast. This may have a more negative impact in a more collectivistic culture (Takeuchi et al., 2015). In this vein, a recent meta-analysis (Kim et al., 2014) confirmed that the relationship between IM and likeability/job performance is stronger in collectivistic countries than in individualistic countries. Thus, future research may explore the effectiveness of exemplification tactics in terms of performance appraisal in a different cultural context.

It would also be convenient to analyze other individual difference variables related to the targets of exemplification attempts. For example, it has been suggested in the context of performance appraisal that rater self-efficacy could influence the effect of IM tactics. Supervisors with low self-efficacy might be more predisposed to subordinates’ ingratiation tactics because they find it difficult to “just say no”, and they may artificially boost performance ratings (Villanova & Bernardin, 1989). From an actor’s viewpoint, there is also evidence that the use of self-focused IM techniques enhances self-efficacy levels, which has been related to employee performance (Aggarwal & Krishnan, 2013).

Furthermore, it would be interesting to address the emotional consequences of the use of exemplification tactics on co-workers. In this sense, it has been noted that self-sacrifice behaviors tactics “may make others around them feel inadequate and guilty” (McGowan & Sekaja, 2022, p. 7). Moreover, recent studies have found that some IM tactics (i.e., self-promotion and ingratiation) moderate the influence of person-organization fit on perceived workplace inclusion (Chen & Tang, 2022). We recommend that future studies focus on the role of exemplification in perceived workplace inclusion.

Finally, future research can examine IM behavior in online contexts. Due to the advancement of information and communication technology and the rapid introduction of digital communication tools as a result of the COVID-19 pandemic, workplace behaviors, including IM, are increasingly implemented through digitalized communication. Technological advancement in the metaverse has the potential to enable more and more organizational behaviors to be conducted in digitalized ways. Although several recent studies have dealt with IM in online contexts, exemplification through online communication has not been explored yet (Al-Shatti & Ohana, 2021). Previous findings on SST suggest that our finding on the moderating effect of age will also hold in online contexts. However, future research can directly examine the effect of “online” exemplification by focusing on employees’ exemplification through digitalized communication.

## Conclusion

Our findings suggest that an employee’s effective use of IM tactics at work depends on the appropriate perception and interpretation of the target’s emotions. In fact, our results support the idea that the use of exemplification tactics may have negative consequences for the user when the actor lacks social skill (i.e., EI). Moreover, the effectiveness of these self-sacrificial behaviors is also a function of the personal characteristics of those at whom these behaviors are directed. Specifically, this study confirms that exemplification is successful when both the EI of the salesperson and the age of the supervisor have high values.

## Appendix (See Table 6)

**Table 6:** Constructs and measures

Constructs standardized factor loadings	Standardized Factor Loadings
*Liking* Source: Wayne and Liden (1995) Composite Reliability = 0.893; AVE = 0.738	
I like them very much as a person	0.843
I think they would make a good friend	0.873
Please rate your subordinate on the degree to which you like each other (1 = dislike each other very much; 7 = like each other very much)	0.860
*Exemplification* Source: Bolino and Turnley (1999) Composite Reliability = 0.857; AVE = 0.601	
Stay at work late so people will know you are hard working	0.649
Try to appear busy, even at times when things are slower	0.824
Arrive at work early to look dedicated	0.804
Come to the office at night or on weekends to show that you are dedicated	0.811
*Emotional intelligence* Source: Wong and Law (2002) Composite Reliability = 0.893; AVE = 0.512	
I always know what my friends are feeling based on their behavior	0.735
I am a good observer of others’ emotions	0.719
I am sensitive to the feelings and emotions of others	0.745
I have a good understanding of the emotions of those around me	0.704
I always set goals for myself and then try my best to achieve them	0.699
I always tell myself I am a competent person	0.763
I am a self-motivated person	0.644
I always encourage myself to try my best	0.713
*Salesperson Performance* Source: Griffin et al. (2007) Composite Reliability = 0.921; AVE = 0.570	
Carried out the core parts of their job well	0.723
Completed their core tasks well using the standard procedures	0.801
Ensured their tasks were completed properly	0.783
Adapted well to changes in core tasks	0.788
Coped with changes to the way they must perform core tasks	0.777
Learned new skills to help them adapt to changes in core tasks	0.775
Initiated better ways of doing their core tasks	0.559
Came up with ideas to improve the way their core tasks are performed	0.653
Made changes to the way their core tasks are performed	0.718
